# Prevention-Focused Care: The Potential Role of Chiropractors in Hong Kong’s Primary Healthcare Transformation

**DOI:** 10.7759/cureus.36950

**Published:** 2023-03-31

**Authors:** Andy Fu Chieh Lin, Christina Cunliffe, Valerie K Chu, Vincent Chan, Albert C Leung, Rick P Lau, Kary K Lam, Jacky C Yeung, Kingsley Leung, Lucina Ng, Eric Chun-Pu Chu

**Affiliations:** 1 Chiropractic and Physiotherapy Centre, New York Medical Group (NYMG), Hong Kong, HKG; 2 Integrative Medicine, McTimoney College of Chiropractic, College of Health, Abingdon, GBR; 3 Executive Board, Chiropractic Doctors Association of Hong Kong, Hong Kong, HKG

**Keywords:** direct primary care, chiropractic management, chiropractic, chiropractic therapy, primary care medicine, prevention in primary care, primary medical care

## Abstract

Hong Kong's healthcare system is moving toward preventive and primary care to address the complicated demands of the aging population. Chiropractic professionals are in an advantageous position to support a prevention-focused strategy by identifying musculoskeletal problems early, reducing risks, and promoting healthy lifestyles. This article examines how the involvement of chiropractors in public health programs could improve population health in Hong Kong and boost primary care. The inclusion of chiropractors in district health centers and other initiatives would offer safer and more cost-effective choices for treating functional problems and chronic pain. Chiropractors should be involved in policymakers' attempts to create a sustainable healthcare system that meets Hong Kong's long-term healthcare requirements.

## Introduction and background

Hong Kong's population is rapidly aging. According to the Census and Statistics Department's projections, the overall population will increase by 7% from 2019 to 2036, driven primarily by growth in the elderly population, which is expected to increase by 82% from 1.32 million to 2.41 million, representing 30% of the total population in 2036 [1]. The current healthcare system focuses on treating acute and severe illnesses, which results in high costs and overburdened hospitals and specialists. This treatment-focused model is unsustainable in the face of ongoing demographic and epidemiological shifts [2]. A more prevention-oriented system with strong primary care is needed to detect and manage conditions early, reduce risk factors, and empower patient self-care [2]. Shifting the focus from treatment to prevention and making greater investments in primary care is critical to addressing Hong Kong's health challenges cost-effectively and sustainably [2]. Hong Kong's healthcare system focuses on secondary and tertiary care, with a dearth of emphasis on preventative and primary care [2]. The evolution of primary healthcare (PHC) in Hong Kong can be traced back to the 1990 "Health for All, the Way Ahead" report, which affirmed the importance of PHC and provided 102 recommendations for its development [2]. Subsequent government consultation documents from 2008 and 2010 outlined a strategy for primary care reform [2]. Throughout successive healthcare reform consultations, enhancing PHC has been a consistent theme and a key point of consensus [2]. According to the PHC Blueprint, there is an urgent need to strengthen primary care to address Hong Kong's aging population and increasing healthcare demands and relieve the burden on the public sector [2]. Greater collaboration between the public and private healthcare sectors has been proposed, enabling a more strategic use of private services for primary care delivery. However, the primary care reform has progressed slowly. Integrating chiropractors and other non-medical practitioners into primary care could help achieve the longstanding goals of high-quality, accessible musculoskeletal care for Hong Kong residents. This article examines how the involvement of chiropractors in public health programs could improve population health in Hong Kong and boost primary care.

## Review

Publicly funded PHC services

Over the years, the Department of Health and Hospital Authority has mostly provided direct services as part of the government's public-funded PHC program [3]. The government has recently started several government-subsidized or public-private partnership (PPP) healthcare programs [3]. The goal is to use the resources of the private healthcare sector to meet the demand for public PHC services and to improve population health. Since 2009, the Elderly Health Care Voucher Scheme has included chiropractors as healthcare practitioners [4]. Chiropractors have not been included in the service model, despite government initiatives to strengthen district-based PHC. Since 2019, the government has committed to establishing district health centers (DHCs) across Hong Kong to enhance multidisciplinary primary care, strengthen PPPs, and shift the focus from treatment to prevention [5]. Operated by non-governmental organizations via government funding, DHCs aim to better support chronic disease management and reduce the burden on specialists and hospitals [5]. However, the DHCs do not include chiropractors. Integrating chiropractic services could allow DHCs to address musculoskeletal and chronic degenerative health needs more effectively and achieve the goals of high-quality, comprehensive primary care.

Chiropractic care is focused on early detection and prevention

Chiropractors focus on the early detection and correction of musculoskeletal dysfunction to prevent progression to more severe conditions [6]. They are trained to identify structural or movement impairment that could lead to injury or pain if left unaddressed [7]. Chiropractic care reduces the risk of sports injuries, decreases the frequency and intensity of headaches, and reduces disability due to back/neck pain [8]. In chronic conditions such as osteoarthritis, chiropractors can stabilize joints and slow degeneration, preventing complications [9]. By detecting and addressing problems early, chiropractors can help minimize pain and disability, enabling patients to engage in daily activities and exercise to improve their overall wellness [10]. Studies in Hong Kong have demonstrated that chiropractic intervention is highly efficacious, with minimal adverse events. According to a study by the Census and Statistics Department in Hong Kong, 53.6% of 33,700 persons who received chiropractic therapy and >90% of patients reported that chiropractic intervention was effective [11]. More than 50% of people had tried other forms of treatment, including physical therapists (18.0%), Chinese medicine practitioners (18.0%), medical specialists (22.2%), and general practitioners (23.7%) [11]. The conservative nature of chiropractic therapy in which manual spinal adjustments were performed in 960,140 therapy visits concluded that the number of severe adverse events was 2.1 per 1000,000 sessions, indicating that such side effects were extremely rare [12]. Given the evidence base, efficacy, and safety profile of chiropractic care, its integration into Hong Kong's healthcare system could benefit patients' musculoskeletal health and health system sustainability.

Chiropractors provide holistic education and counseling

Chiropractors in Hong Kong provide counseling on lifestyle factors that significantly impact health [13]. This includes advice on proper ergonomics, therapeutic exercises, a healthy diet, weight management, smoking cessation, and stress reduction [13]. These behavioral elements are important in preventing injury/illness and managing chronic conditions [14]. For example, ergonomic changes can reduce musculoskeletal pain caused by desk jobs; exercise helps control weight and blood glucose levels; nutritious eating prevents heart disease; and stress management decreases inflammation and anxiety/depression. Lifestyle education is critical for patients to participate in their wellness and take a preventive, proactive approach to health that aligns with the goals of a prevention-focused healthcare system [15]. Chiropractors use a whole-body approach, recognizing the interconnectedness between the musculoskeletal, nervous, and overall health systems [16]. Hong Kong chiropractors understand how imbalances in the spine or joints, including scoliosis, can affect functions elsewhere in the body [17-24]. This holistic perspective is essential in preventive care, as it helps patients understand how various lifestyle, biological, and environmental factors influence their health in interconnected ways [13]. With this insight, patients are empowered to act on the root causes of illness or pain and not just treat symptoms. A holistic view of health and wellness is critical for a preventive healthcare system aimed at comprehensive long-term well-being and not just treating isolated conditions.

Role of chiropractors in prevention-focused systems internationally

Chiropractors are integrated into public healthcare systems in countries such as North America and Europe, prioritizing prevention and primary care [25]. For example, chiropractors in the UK can work within the National Health Service, which provides early intervention for musculoskeletal complaints and chronic conditions such as osteoarthritis and back pain [26]. In Norway, one-third of women and a quarter of men sought PHC services for musculoskeletal problems, whereas 5-9% used chiropractors, physiotherapists, or a combination of providers [27]. Studies have shown that chiropractic care reduces disability and healthcare costs in these systems while generating high patient satisfaction [28]. In Denmark, chiropractors work alongside general practitioners at public clinics, advising patients on self-care for pain and referring to those who need more intensive rehabilitation [29]. The integration of chiropractors into prevention-focused healthcare systems increases access to conservative and lifestyle-oriented care, eases the burden on doctors, and achieves good patient outcomes at lower costs. Hong Kong can learn valuable international lessons by integrating chiropractors into its healthcare system. Chiropractors should be incorporated into DHCs and public clinics to provide conservative musculoskeletal care and lifestyle education. They can help manage chronic musculoskeletal conditions and provide preventative care. This will increase access to drug-free pain management and prevention strategies. Chiropractors can also help achieve the goals of DHCs by empowering patients to participate in their wellness through self-care. With training for chiropractors and medical doctors in each other's roles, collaborative models have become effective in other systems. Integrating chiropractors into Hong Kong's healthcare system could provide affordable and sustainable musculoskeletal and wellness-focused care, relieve the burden on hospitals, and complement the government's vision of a more preventive and primary care-oriented model.

Strategy to improve health status, provide healthcare services, and establish a sustainable healthcare system

To achieve a sustainable community-based PHC system for Hong Kong, the government blueprint recommends developing district networks, strengthening governance, consolidating resources, reinforcing human power, and improving data systems [2]. Here, we discuss how incorporating chiropractors into Hong Kong's envisioned primary care system will increase access to conservative early intervention and prevention strategies for musculoskeletal health, help limit costs, and support aging.

Develop a Community-Based Primary Healthcare System

To realize the government's goal of a district-based system centered on integrated and coordinated care [5], chiropractors should be included in Hong Kong's PHC strategy. Chiropractors could support the "family doctor for all" approach by offering drug-free pain care and promoting long-term well-being as conservative musculoskeletal specialists. They might work with medical professionals to co-manage chronic diseases and help with common concerns such as headaches and back/neck discomfort. Through protocol-guided collaborative treatment, chiropractors can improve the capacity of DHCs and family physicians to manage chronic diseases. Its inclusion would also improve the primary care system's comprehensive preventive approach by enabling patients to play an active role in their own health through self-care practices [5]. Access to non-pharmacological care choices would be improved, and the blueprint's emphasis on horizontal/vertical integration and health/social partnership would be achieved by integrating chiropractors into district-based networks and chronic illness pathways [5].

Strengthen PHC Governance

Chiropractors should be included in developing and implementing policies to improve PHC governance in Hong Kong [2]. A more holistic approach to policymaking that balances resources and attention across primary, secondary, and tertiary care could be facilitated by involving chiropractors as stakeholders [2]. Chiropractic professionals may provide suggestions for expanding access to conservative treatment alternatives. Thus, chiropractors should participate in initiatives to improve primary care inter-organizational collaboration, integrated care delivery, and performance monitoring at the implementation level [6]. Evaluating the effects of chiropractic care on outcomes and costs would be possible if data collection and transparency regarding its functions and contributions were standardized. Chiropractors should contribute to their clinical skills to promote the reforms required for high-quality collaborative PHC.

Consolidate PHC Resources

To consolidate the resources for a stronger PHC system, chiropractors should be integrated to maximize the use of existing funds. Despite requests to promote primary care, Hong Kong allocated more resources to secondary and tertiary care [2]. The integration of chiropractors and other primary care providers may be facilitated by the redistribution of money from downstream care and a decrease in the usage of hospitals and specialists [2]. Integrating chiropractors could decrease referrals to specialists and scans/surgeries for common problems, such as back/neck pain, given their focus on conservative musculoskeletal therapy. Chiropractors should expand the clinical capabilities of primary care networks and maximize fresh investments. By controlling costs through earlier intervention and self-care instruction rather than more expensive downstream therapies when diseases become severe, the inclusion of chiropractors would aid in achieving financial sustainability [2].

Reinforce PHC Manpower

Chiropractors should be integrated into the workforce to reinforce PHC personnel. Chiropractors are educated and trained to diagnose and conservatively manage different conditions [30], significantly burdening Hong Kong's healthcare system. By including chiropractors in primary care teams, their specialized skills and experience can strengthen the range of conditions that can be effectively addressed in community settings. Chiropractors are also trained to collaborate with other healthcare professionals, e.g., medical doctors, physiotherapists, and nurses [6]. Establishing interprofessional education and promoting team-based care delivery models would help chiropractors meet patients' needs collaboratively [6]. Integrating chiropractors would increase access to a wider range of primary care services comprising most of the existing healthcare workforce.

Improve Data Connectivity and Health Surveillance

Chiropractors should be incorporated into digital health networks and data-gathering platforms to improve data connections and health surveillance, including electronic health record-sharing systems (eHealth) [31]. Including chiropractors would enable the detection and management of chronic musculoskeletal diseases, which account for a sizable amount of healthcare spending [7]. The success of conservative techniques and follow-up information on common symptoms, such as headaches and back/neck discomfort, can all be provided by chiropractors [13, 14, 16]. A more complete picture of the population's health requirements and the effects of integrated primary care can be obtained from these data. By integrating chiropractors into health information systems, we can improve the prevention and management of illnesses that fall under the purview of chiropractic practice. It would also make it possible to compare chiropractors' contributions to cost-cutting and outcome improvement with those of other healthcare providers. In conclusion, incorporating chiropractors into Hong Kong's PHC system is vital for successfully addressing the challenges associated with an aging population. Strong district-based primary care is essential for treating patients, preventing life-threatening illnesses, and advancing public health, as emphasized by the pandemic. The integration of chiropractors would not only relieve hospitals from the burden of managing common musculoskeletal and degenerative conditions but also promote self-care and enable the monitoring of chiropractic outcomes and costs compared to other care models. This would lead to continuous improvement of the PHC system in Hong Kong through outcome evaluation. Although the study has limitations, such as a lack of methodology, data sources, analysis, and limited contextual information about the role of chiropractors in Hong Kong, the finding effectively highlights the potential for chiropractors to improve public health through early identification, risk reduction, and holistic lifestyle teaching.

## Conclusions

In Hong Kong, chiropractors are in an advantageous position to support a healthcare system that emphasizes prevention. Chiropractors can aid in the early detection of health concerns through musculoskeletal evaluation. They can offer advice on how to lower the chance of developing common ailments, such as arthritis and back discomfort. To promote long-term health, chiropractors place a strong emphasis on holistic lifestyle education. A trend toward preventative care and strengthening Hong Kong's healthcare system would be supported by including chiropractors in primary care programs. Policymakers may provide citizens with safer and more cost-effective options for treating functional problems and chronic pain by including chiropractors in DHCs and other public health initiatives. This will assist in addressing the intricate and expanding health demands of an aging population. Chiropractors can potentially improve public health through early identification, risk reduction, and holistic lifestyle teaching. Policymakers in Hong Kong should take advantage of this potential as they seek to develop a long-term healthcare system that includes chiropractors in the interest of patients.
